# Case Report: The application of metagenomic next generation sequencing in diagnosing fungal malignant external otitis: a report of two cases

**DOI:** 10.3389/fcimb.2023.1236414

**Published:** 2023-11-20

**Authors:** Qi Wang, Rui Hu, YiFan Zhu, WenQing Zhu, Hua Jiang

**Affiliations:** Department of Otolaryngology, The Second Affiliated Hospital Zhejiang University School of Medicine, Hangzhou, China

**Keywords:** fungal malignant external otitis, *Aspergillus*, metagenomic next-generation sequencing, case report, voriconazole

## Abstract

**Background:**

Most of malignant external otitis (MEO) cases reported in the literature are attributed to *Pseudomonas aeruginosa*. Fungal infections in MEO are also likely but extremely rare. And conventional microbiology tests is difficult to diagnose.

**Case description:**

Two patients were diagnosed with Fungal malignant external otitis (FMEO) due to *Aspergillus* by metagenomic Next-Generation Sequencing (mNGS) and recovered after comprehensive treatment including operation and voriconazole. The antifungal treatment was delayed due to repeated cultures of secretions being negative and pathological examination showed granulation tissue proliferation with extensive neutrophil infiltration.

**Conclusion:**

mNGS might be helpful for patients suspected with FMEO, especially when conventional microbiology tests were negative.

## Introduction

MEO is an aggressive and potentially life-threatening infection. The main feature is the diffusion from the external auditory canal to adjacent anatomical structures, including soft tissue, cartilage, and bones (Vennewald and Klemm, 2010). Approximately 95% of MEO cases reported in the literature are attributed to *Pseudomonas aeruginosa* (Carfrae and Kesser, 2008). Fungal infections in MEO are also likely but extremely rare, which could be caused by *Aspergillus* and *Candida* species. Risk factors include uncontrolled diabetes and immunocompromised patients. Culture of ear canal secretions and pathological examination may fail to identify fungal infections. On the other hand, mNGS has been increasingly used in the diagnosis of infectious diseases, particularly when conventional tests were negative.

Here we report two cases of FMEO due to *Aspergillus* infections, in which the diagnosis was eventually established with the help of mNGS. Microbiological culture of ear canal secretions and pathological examination were negative and antifungal treatment was delayed.

## Case presentation

### Case 1

**Figure 1:**
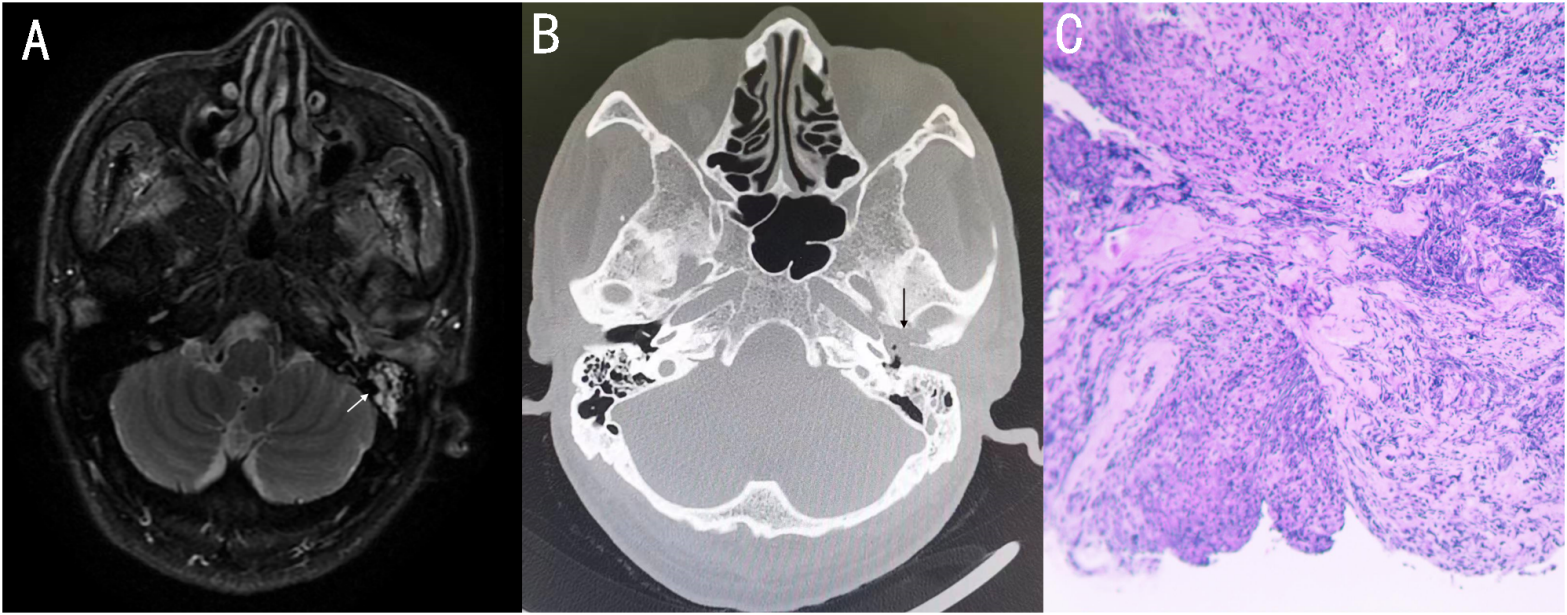
**(A)** MRI showed thickening and strengthening of the wall of the left external auditory canal cavity and left mastoiditis. **(B)** Axial CT showing soft tissue involvement of the left external and the middle ear, with external auditory canal - tympanum wall bone erosion. **(C)** 11-10 Postoperative pathology: granulation tissue proliferation with extensive neutrophil infiltration.

**Figure 2:**
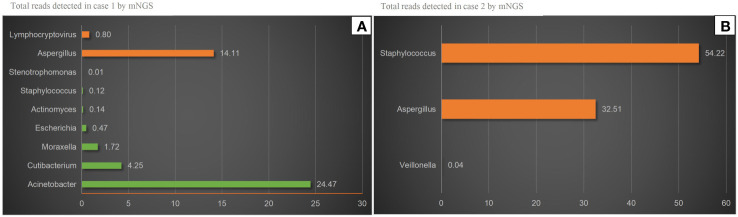
mNGS results. **(A)** Aspergillus and Lymphocryptovirus were detected in case 1 by mNGS. Aspergillus was accounting for 14.11% of all microbial reads. The others were considered Probable normal flora. **(B)** Aspergillus and Staphylococcus were detected in case 2 by mNGS. Aspergillus was accounting for 32.51% of all microbial reads. Veillonella was considered Probable normal.

**Figure 3:**
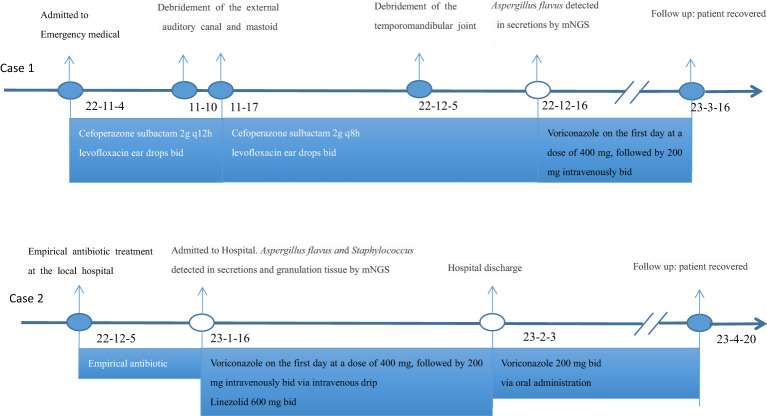
The treatment timeline of the patient.

On November 4, 2022, a 73-year-old male with diabetes presented with otalgia for 1 month. The otalgia was severe and the patient could not sleep. Analgesics can only relieve the symptom for 2-3 hours. Upon physical examination, there was suppurative mucous discharge and granulation tissue in the left ear canal. Culture of secretion yielded no growth of bacteria or fungi. Examination of blood showed a CRP of 130 mg/l and WBC of 12.45 x 10^9^/l. MRI revealed thickening and strengthening of the wall of left external auditory canal cavity and left mastoiditis (**Figure 1**). CT scan confirmed the lesion of the temporal bone external auditory canal-tympanic wall (**Figure 1**). The patient was treated with cefoperazone sulbactam 2g (q12h) and levofloxacin ear drops, and sodium bicarbonate solution for ear canal irrigation. However, no improvement of symptoms was seen after one week. Debridement of the external auditory canal and mastoid were carried out to remove the infected and necrotic tissues on November 10^th^ and 17^th^. Pathological examination showed granulation tissue proliferation with extensive neutrophil infiltration (**Figure 1**). As a result, ceftazidime was administered (2g, q8h) but again failed to relieve the symptoms and the patient developed facial paralysis. Debridement of the temporomandibular joint was performed on December 5^th^. During the operation, a large amount of inflammatory granulation tissue in the bilateral plate area, bone defect in the anterior wall of the external auditory canal were found. The symptoms persisted after surgery and mNGS was performed on granulation tissue on December 16^th^, which reported *Aspergillus flavus* (**Figure 2**). Voriconazole was administered intravenously twice on the first day at a dose of 400 mg, followed by 200 mg intravenously (BID). Following one month of antifungal therapy, the patient’s earache showed significant clinical improvement. After discharge, voriconazole was taken orally (200 mg twice a day for 2 months) and the patient recovered despite that facial paralysis persisted. The entire treatment process is summarized in **Figure 3**.

### Case 2

**Figure 4:**
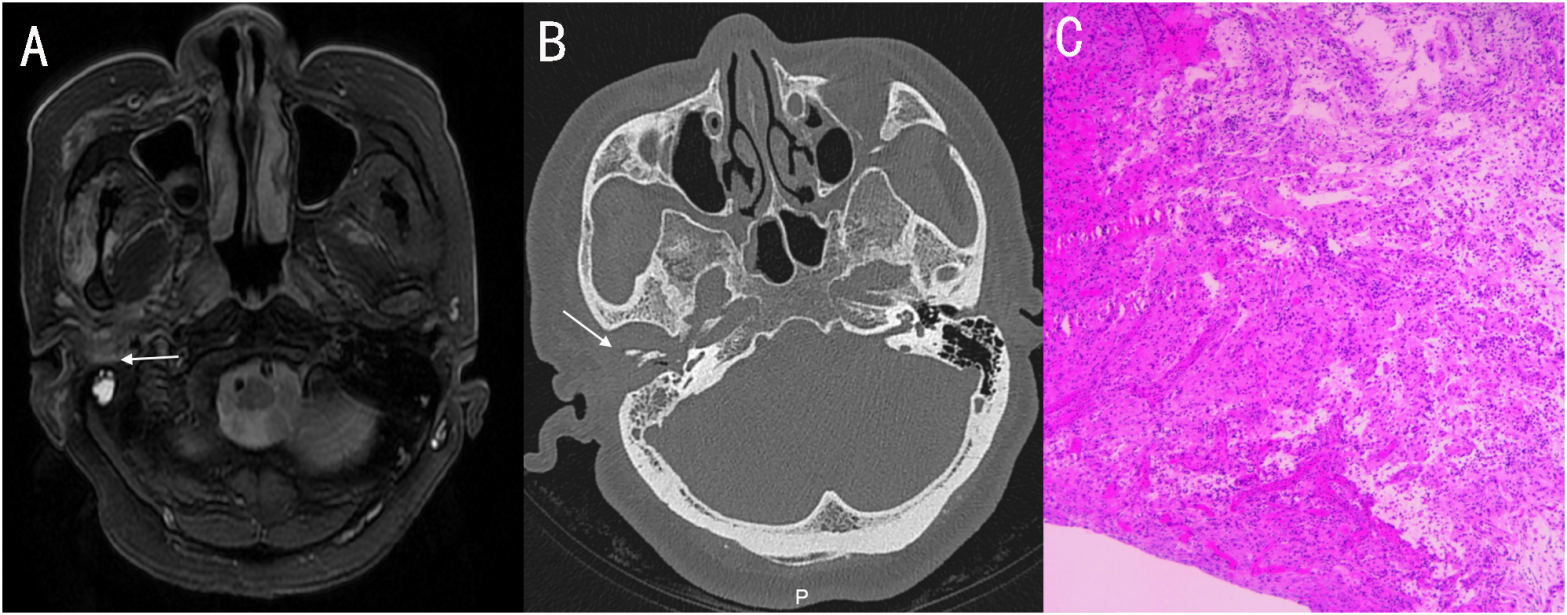
**(A)** MRI showed irregular tissue masses in the external auditory canal, with unclear boundaries. **(B)** Axial CT showing soft tissue involvement of the right external, with temporomandibular joint and eustachian tube bone erosion. **(C)** pathology: granulation tissue proliferation with extensive neutrophil infiltration.

A 56-year-old female with diabetes presented with otalgia and otorrhea for 40 days. Repeated cultures of secretions were negative, and the symptoms were not relieved after empirical antibiotic treatment at the local hospital. Physical examination showed suppurative mucous discharge and granulation tissue in the right ear canal, as well as mild facial paralysis. CT scan showed the lesion in the temporal bone external auditory canal - tympanic wall (**Figure 4**). MRI showed irregular tissue masses in the external auditory canal, with unclear boundaries (**Figure 4**). Pathological examinations showed granulation tissue (**Figure 4**). mNGS on ear canal secretions and granulation tissue was performed immediately upon admission, and the results showed presence of *Aspergillu*s *flavus* and *Staphylococcus* (**Figure 2**). Voriconazole was administered at a dosage of 400 mg intravenously twice for the first day, followed by 200 mg intravenously (BID). Linezolid was taken orally (600 mg twice a day for two weeks). Two weeks later, the patient was discharged and switched to oral voriconazole, which lasted for 3 months. The patient fully recovered after the treatment. The entire treatment process is summarized in **Figure 3**.

## Discussion

FMEO occurs in immunocompromised patients, such as AIDS, acute leukemia, uncontrolled diabetes mellitus. The first case of FMEO was described in 1985 in a 68-year-old patient with recurrent acute myeloid leukemia (Petrak et al., 1985).

Clinical signs of FMEO include severe otalgia, purulent otorrhea, granulation and neurologic deficits (especially facial nerve involvement), previously diagnosed otitis externa not responsive to therapy.

The progression of the FMEO has been divided into 3 clinical stages (Bovo et al., 2012). Stage I: infection of the external auditory canal and adjacent soft tissues with severe pain, with or without facial nerve palsy. Stage II: extension of infection with osteitis of skull base and temporal bone, or multiple cranial nerve neuropathies. Stage III: intracranial extension with meningitis, epidural empyema, subdural empyema or brain abscess. Meningitis, large vessel septic thrombophlebitis or rupture, septicemia, aspiration pneumonia due to vagal paralysis, and cerebrovascular accident are the most frequent causes of death. The examination of invasive fungal infection includes direct microscopic examination of fungi, fungal culture and identification, serological examination, histopathological examination, and Polymerase chain reaction(PCR). Fungal culture and antifungal susceptibility test are helpful to guide clinical treatment. However, conventional culture-based techniques have a typically low detection rate and culture is time consuming (Weiss et al., 2019). Direct microscopic examination of fungi is a simple and rapid method. But *Aspergillus* is also frequently isolated from external auditory canal smears and diagnosis of FMEO should be based on histopathologic confirmation on deep tissue biopsy (Martínez-Berriotxoa et al., 1997). A deep tissue specimen is necessary to diagnose the fungi: this could reduce the risk of contamination or isolation of pathogenic saprophytes (Tuzcu et al., 2006). Tissue biopsies were taken from the external ear canal revealed nonspecific chronic inflammation in our cases. The advantages of mNGS sequence-based detection of microbes associated with a disease state include high throughput evaluation that can accommodate many samples at once compared to traditional PCR-based detection, the quantity of data provided by sequence is potentially much higher because bacterial, fungal, and viral community members can be detected simultaneously, and the reliability of sequence data surpasses that of traditional morphological and physiological assays for microbial identification (Jiang et al., 2021). The cost of mNGS is expensive, which is not conducive to widespread promotion. Compared with traditional cultivation methods, mNGS cannot perform drug sensitivity and is not conducive to drug screening. mNGS might be recommended for high-risk patients, who can benefit from early diagnosis and treatment. At the same time, it is recommended to use deep tissue for mNGS sampling materials.

Treatment for FMEO usually involves extensive surgical debridement, intensive long-term antifungal treatments, aggressive control of diabetes, and improvement of immunocompetency when possible (Halsey et al., 2011). However, there are neither guidelines nor definite recommendations with regards to surgical treatment of FMEO. Some authors emphasize that extensive surgery may be counterproductive because of the risk of exposing healthy bone to infection (Carfrae and Kesser, 2008). Amphotericin B and itraconazole were favored for treatment of FMEO in the earlier case reports, whereas voriconazole has played a role in the therapy of the majority of reported cases since 2008 (Walton and Coulson, 2014). Voriconazole is currently recommended as first-line treatment in cases of invasive aspergillosis (Walton and Coulson, 2014). Treatment failure may also be due to discontinuation or reduction of treatment due to antifungal toxicity (Perrine et al., 2009). In the review by M. Mion et al. (2015), 64% of patients were treated with a single antifungal agent (Voriconazole or amphotericin B) for an average course of 178.4 days. The proportion of antifungal combination therapy was 36% (9/25), and the mean treatment time was 151.62 days. The rate of surgical debridement was 56%. Fully recovered patients accounted for 60% (15/25). The most common adverse effects were renal impairment, increased blood urea nitrogen and creatinine, decreased creatinine clearance and renal failure. Voriconazole is currently considered the first-line treatment option for invasive aspergillosis with good clinical and biological tolerability. Voriconazole showed better fungicidal activity against *Aspergillus fumigatus* than itraconazole (Lewis et al., 2005). Its oral bioavailability is greater than 90%, which means that patients treated with this drug do not require long-term hospitalization (Muijsers et al., 2002). Hsu-Chueh Ho et al (Hsu-Chueh. et al., 2014). reported three cases of FMEO treated by Voriconazole. One required surgical detoxification after discontinuation, and two recovered after conservative treatment. The main side effects of voriconazole were liver damage, therefore, liver function should be monitored every 2 weeks. Concentrations of liver markers can be used to assess the dosage to minimize side effects.

The diagnosis and treatment of FMEO in our report serves as a reference for clinical management of these patients. For high-risk patients suspected of FMEO, mNGS tests are recommended when conventional microbiological tests are negative. The clinician’s experience is important for early diagnosis, and the key to its treatment is antifungal therapy, surgical debridement, and primary disease control.

## Data availability statement

The raw data supporting the conclusions of this article will be made available by the authors, without undue reservation.

## Ethics statement

The studies involving humans were approved by Human Research Ethics Committee of the Second Affiliated Hospital of Zhejiang University School of Medicine. The studies were conducted in accordance with the local legislation and institutional requirements. The participants provided their written informed consent to participate in this study. Written informed consent was obtained from the individual(s) for the publication of any potentially identifiable images or data included in this article. Written informed consent was obtained from the participant/patient(s) for the publication of this case report.

## Author contributions

QW: patient management, data collection, and writing original draft. RH and YZ: patient management and data collection. WZ: review and modify the article. HJ: review and modify the article. All authors contributed to the article and approved the submitted version.
