# Supplementary material for: Case Report: The application of metagenomic next generation sequencing in diagnosing fungal malignant external otitis: a report of two cases
Source: Front Cell Infect Microbiol. 2023 Nov 20;13:1236414. doi: 10.3389/fcimb.2023.1236414 (PMC10694228; doi:10.3389/fcimb.2023.1236414)
Supplement: Supplementary file 1 [file DataSheet_1.pdf]

Case 1

Specimen type: granulation

Test result

Pathogenic microor: *Aspergillus flavus/Aspergillus oryzae, Lymphocryptovirus* (human herpesvirus type 4 (EB virus))

Probable normal flora: Acinetobacter jeunei, Propionibacterium acnes, Moraxella Oslo, Escherichia coli, Actinobacteria Neisseria, Staphylococcus epidermidis

Genus	relative abundance %
Acinetobacter	24.47
Cutibacterium	4.25
Moraxella	1.72
Escherichia	0.47
Actinomyces	0.14
Staphylococcus	0.12
Stenotrophomonas	0.01
Aspergillus	14.11
Lymphocryptovirus	0.8

Case 2

Specimen type: granulation and secretion

Test result

Pathogenic microor: Aspergillus flavus/Aspergillus oryzae, Staphylococcus aureus
Probable normal flora: *Veillonella parvula*

Genus	relative abundance %
Veillonella	0.04
Aspergillus	32.51
Staphylococcus	54.22
